# The AMPK Activator A769662 Blocks Voltage-Gated Sodium Channels: Discovery of a Novel Pharmacophore with Potential Utility for Analgesic Development

**DOI:** 10.1371/journal.pone.0169882

**Published:** 2017-01-24

**Authors:** Marina N. Asiedu, Chongyang Han, Sulayman D. Dib-Hajj, Stephen G. Waxman, Theodore J. Price, Gregory Dussor

**Affiliations:** 1 University of Arizona, Department of Pharmacology, Tucson, Arizona, United States of America; 2 University of Texas at Dallas, School of Behavioral and Brain Sciences, Richardson, Texas, United States of America; 3 Yale School of Medicine, Department of Neurology, Center for Neuroscience and Regeneration Research, and Veterans Administration Connecticut Healthcare System, Rehabilitation Research Center, West Haven, Connecticut, United States of America; University of Kentucky Medical Center, UNITED STATES

## Abstract

Voltage-gated sodium channels (VGSC) regulate neuronal excitability by governing action potential (AP) generation and propagation. Recent studies have revealed that AMP-activated protein kinase (AMPK) activators decrease sensory neuron excitability, potentially by preventing sodium (Na^+^) channel phosphorylation by kinases such as ERK or via modulation of translation regulation pathways. The direct positive allosteric modulator A769662 displays substantially greater efficacy than other AMPK activators in decreasing sensory neuron excitability suggesting additional mechanisms of action. Here, we show that A769662 acutely inhibits AP firing stimulated by ramp current injection in rat trigeminal ganglion (TG) neurons. PT1, a structurally dissimilar AMPK activator that reduces nerve growth factor (NGF) -induced hyperexcitability, has no influence on AP firing in TG neurons upon acute application. In voltage-clamp recordings, application of A769662 reduces VGSC current amplitudes. These findings, based on acute A769662 application, suggest a direct channel blocking effect. Indeed, A769662 dose-dependently blocks VGSC in rat TG neurons and in Na_v_1.7-transfected cells with an IC_50_ of ~ 10 μM. A769662 neither displayed use-dependent inhibition nor interacted with the local anesthetic (LA) binding site. Popliteal fossa administration of A769662 decreased noxious thermal responses with a peak effect at 5 mins demonstrating an analgesic effect. These data indicate that in addition to AMPK activation, A769662 acts as a direct blocker/modulator of VGSCs, a potential mechanism enhancing the analgesic property of this compound.

## Introduction

Pain hypersensitivity resulting from injury or disease is often paralleled by increased excitability of peripheral sensory neurons in either the dorsal root ganglion (DRG) or trigeminal ganglion (TG) [1,2]. Voltage-gated Na^+^ channels (VGSCs) regulate the generation and propagation of action potentials in excitable tissues and thus are critical determinants of excitability in primary sensory neurons [3,4]. The identification of activity-dependent changes in VGSCs in preclinical models [5,6] and the linkage of mutations in VGSCs to genetic pain disorders in humans [7,8,9,10,11] illustrates a key role for these channels in pain hypersensitivity in animals and humans. Therefore, these channels are key drug targets for the development of novel analgesics.

Compelling evidence from human pain conditions implicates a critical key role of VGSCs particularly, Na_v_1.7, in determining pain sensitivity. Na_v_1.7 is a threshold channel known to amplify generator potentials toward threshold for action potential firing [12,13]. A complete loss-of-function in Na_v_1.7 results in insensitivity to pain [14,15,16] whereas a gain-of- function causes erythromelalgia and paroxysmal extreme pain disorder [8]. Several lines of evidence also implicate VGSCs in acquired pain states. Inhibiting VGSCs with small molecules reduces sensory neuron excitability [17,18,19]. Further, block of the channel by small molecules shows that Na_v_1.7 contributes to the major TTX-sensitive Na^+^ current in both small-diameter mouse and human DRG neurons [20,21,22]. In mice, the genetic deletion of VGSCs produces striking decreases in normal and pathological pain [23,24,25,26]. In addition, non-selective VGSCs inhibitors reduce allodynia in preclinical neuropathic pain models [5,6,27,28,29,30].

The extracellular signal-related kinase1/2 (ERK1/2) modulates Na_v_1.7 via phosphorylation on specific residues on an intracellular loop of the channel. The phosphorylation of Na_v_1.7 by ERK1/2 mediates an increase in action potential firing and a decrease in latency to first action potential [31]. A key kinase that negatively regulates ERK activity is the energy-sensing kinase, AMP-activated protein kinase (AMPK) [32,33,34]. AMPK is a ubiquitous kinase that acts as an endogenous cellular sensor of AMP and ADP levels under low cellular energy conditions [32]. Activation of AMPK with pharmacological tools such as A769662 and 5-Aminoimidazole-4-carboxamide ribonucleotide (AICAR) results in inhibition of signaling through anabolic pathways [35] like ERK. AMPK activators, including A769662, decrease DRG and TG neuron excitability as well as hyperexcitability induced by pain promoting endogenous mediators such as nerve growth factor (NGF) [33]. Interestingly, A769662 possesses greater efficacy in reduction of DRG and TG neuron excitability than other AMPK activators [33]. A possible explanation for this effect is a direct action on VGSCs. Herein we tested this hypothesis, and show that acute application of A769662 onto TG neurons abolishes action potential firing and reduces VGSC current amplitude in a dose-dependent manner. Inhibitory actions are also observed in Na_v_1.7-transfected HEK293 cells. Hence, in addition to AMPK activation, A769662 acts as a direct blocker/modulator of VGSCs, including Na_v_1.7, and ameliorates pain behavior in an animal model.

## Materials and Methods

### Animals

Adult male Sprague-Dawley rats (225–250g) purchased from Harlan were used for the patch-clamp electrophysiology and behavior studies. All animal procedures were approved by the IACUC at University of Arizona and University of Texas at Dallas and were in compliance with NIH and International Association for the Study of Pain guidelines.

### Cell lines and experimental compounds

Na_v_1.7-transfected human embryonic kidney 293 (HEK293) cells were described previously [12,36,37]. Stock solutions of A769662 (LC Laboratories, Woburn, MA), Resveratrol (Cayman Chemicals, Ann Arbor, MI) and PT1 (Tocris, Minneapolis, MN) were made in DMSO and diluted in external solution bringing the final concentration of DMSO to <1%. For in vivo experiments, A769662 and PT1 were diluted in 100 mM PBS. Metformin was purchased from LKT Laboratories (St. Paul, MN) and stock solutions were made in double deionized water.

### Cell culture

Rats were anesthetized with 5% isoflurane (Vedco Inc., St. Joseph, MO) and sacrificed by decapitation. TGs were dissected and placed in ice-cold Hanks balanced-salt solution (divalent free). Ganglia were cut into small pieces and incubated for 25 min in 20 U/ml Papain (Worthington, Lakewood, NJ) followed by 25 min in 3 mg/ml Collagenase Type II (Worthington). After trituration through a fire-polished Pasteur pipette, cells were plated on poly-D-lysine and laminin (Sigma, St. Louis, MO)—coated plates. Cells were allowed to adhere for several hours at room temperature in a humidified chamber and then nourished with Liebovitz L-15 medium (Life Technologies, Grand Island, NY) supplemented with 10% fetal bovine serum (FBS), 10 mM glucose, 10 mM HEPES and 50 U/ml penicillin/streptomycin. To assess NGF-induced excitability, TG neurons were treated with 50 ng/ml NGF (Millipore). In other experiments assessing direct VGSC modulation, NGF was not used. Cells were used within 24 hrs post plating. HEK293 cells were grown in Dulbecco’s modified Eagle’s medium-F12+L-Glutamine (Life Technologies) supplemented with 10% FBS and 0.6 mg/ml Geneticin (Invitrogen, Carlsbad, CA), plated, kept at 37° C, and split before reaching 75% confluence. For transient transfection, the plasmids carrying wild-type hNav1.7 or F1737A/Y1744A mutant channels with impaired local anesthetic binding site (McCormak et al PNAS 2013) were cotransfected with enhanced GFP (chanel: EGFP ratio 4:1) into HEK293 cells using LipoJet^TM^ transfection reagents (SignaGen^®^ Laboratories). After transfection, cells were grown in at 37°C for 48 h before electrophysiological recordings.

### Electrophysiology

Whole cell patch-clamp experiments were performed on isolated rat TG neurons *in vitro* and Na_v_1.7-transfected HEK293 cells using a MultiClamp 700B (Axon Instruments) patch-clamp amplifier and PClamp 9 acquisition software (Axon Instruments). Recordings were sampled at 20 kHz and filtered at 1 kHz (Digidata 1322A, Axon Instruments). Pipettes (OD: 1.5 mm, ID: 0.86 mm, Sutter Instrument) were pulled using a P-97 puller (Sutter Instrument) and heat polished to 1.5–4 MΩ resistance using a microforge (MF-83, Narishige). Series resistance was typically < 5 MΩ and was compensated 60–80%. The seal stability was examined and cells with an initial seal resistance of <5 GΩ, membrane blebs, or high leak currents (> 0.2 nA) were not considered for analysis. Data were analyzed using Clampfit 10 (Molecular Devices and Origin 8 from OriginLab). For neuronal VGSC current recordings, pipette solution contained (in mM) 120 CsCl, 10 EGTA, 2 MgCl_2_, 5 NaCl, 10 HEPES, 2 CaCl_2_, pH 7.3 (adjusted with N-methyl glucamine) and osmolarity was 320 mosM. External solution contained (in mM) 95 Choline, 20 Tetraethyl ammonium (TEA), 20 NaCl, 2 CaCl_2_, 1 MgCl_2_, 10 HEPES, 5 KCl, 0.1 CdCl_2_, 0.1 NiCl_2_, pH 7.3 (adjusted with N-methyl glucamine) and osmolarity was 320 mosM. For HEK cell recordings, pipette solution contained (in mM) 140 CsF, 1 EGTA, 10 NaCl, 10 HEPES, pH 7.3 (adjusted with N-methyl glucamine) and osmolarity was 320 mosM. External solution contained (in mM) 140 NaCl, 1 CaCl_2_, 1 MgCl_2_, 10 HEPES, 3 KCl, 10 Glucose, pH 7.3 (adjusted with N-methyl glucamine) and osmolarity was 320 mosM. The osmolarity of each solution was adjusted with sucrose solution.

For current clamp recordings, the resting membrane potential (RMP) was examined and neurons with RMP more positive than -45mV were excluded. The average RMP of the recorded cells was -60.53 ±0.36 mV. The RMP was recorded 1–3 min after achieving whole-cell configuration. Action potentials were elicited by injecting slow ramp currents from 0.1 to 0.7 nA with Δ = 0.2 nA over 1 sec to mimic slow depolarization. The pipette solution for ramp current recordings contained (in mM) 140 KCl, 11 EGTA, 2 MgCl_2_, 10 NaCl, 10 HEPES, 1 CaCl_2_ pH 7.3 (adjusted with N-methyl glucamine), and was ~ 320 mosM. External solution contained (in mM) 135 NaCl, 2 CaCl_2_, 1 MgCl_2_, 5 KCl, 10 Glucose, 10 HEPES, pH 7.4 (adjusted with N-methyl glucamine), and was ~ 320 mosM. Small diameter TG neurons with an average capacitance of 18 pF (range 15–22 pF) were used for all experiments. All recordings were done at room temperature.

For voltage-clamp studies in HEK293 cells transiently expressing hNa_v_1.7 channels, whole-cell patch-clamp recordings were performed at room temperature 48 h after transfection using an EPC-10 amplifier (HEKA Electronics). Currents were elicited from a holding potential of -120 mV, filtered at 5 kHz, and acquired at 50 kHz using Patchmaster (HEKA Electronics). Voltage errors were minimized using 90% series resistance compensation in all recordings. The liquid junction potential was not corrected. The compositions of recording bath solution and pipette solution were the same as the solutions for recording with Na_v_1.7 in stable cell lines described above. Electrophysiological data were analyzed using Fitmaster (HEKA Electronics) and Origin 8.5.1 (Microcal). Drug solutions were made fresh before recordings and applied through a gravity-driven system with perfusion pencil (Automate Scientific) allowing a rapid perfusion of the recording chamber. During the recording, the cells were continuously perfused with test solution.

### *In vivo* assessment of nerve conduction block using the hargreaves test

Rats were placed in Plexiglas boxes on a clear glass plate for habituation for 30 min. A radiant heat source was directed onto the plantar surface of the left hind paw. A motion detector halted both heat lamp and timer when the paw was withdrawn. Baseline thermal latencies were established [38]. Nerve conduction block was assessed by injecting A769662 (30μg, 100μg, 300μg) or PT1 (300μg) into the popliteal fossa in a volume of 200 μl under light isofluorane anesthesia. Anesthesia was induced with 4% isoflurane and maintained at 2%. The breakdown of the duration of time for the entire procedure is as follows: from anesthesia induction to popliteal fossa injection (40.2 ±1.6 sec), post injection with anesthesia to awake and moving (55.9 ± 4.5 sec) and the time of start of behavior testing was (152.1±10.4 sec) after anesthesia induction. Thermal latencies were then assessed at the indicated time points over a total of 25 min. Animals were randomized to groups (4–7 rats). A maximum cutoff was set at 30 sec to prevent tissue damage.

### Rotarod test

The motor performance of the rats was assessed using the rotarod as previously performed [39]. Rats were trained in three consecutive sessions to stay on the rod and reach a cut off time of 180 sec at constant rate of rotation of 8 revolutions per min (IITC Rotarod Series 8, IITC inc.,Woodland Hills, CA). The rats were tested ~2.5 mins after isoflurane administration which is about 2.5 mins before the 5 minute time point in the thermal latency studies to ensure the absence of any impact on motor performance.

### Statistics

All data are presented as mean ± standard error of the mean (SEM). Graph plotting and statistical analysis used Graphpad Prism Version 5.03 (Graph Pad Software, Inc. San Diego, CA, USA). Statistical evaluation was performed by two-way analysis of variance (ANOVA), followed by Bonferroni’s post-hoc tests for multiple comparisons or by unpaired student’s t-test for pair-wise comparisons. Dose-response calculations were done using non-linear regression with variable slope (four parameters). The *a priori* level of significance at 95% confidence level was considered at *p* < 0.05. Raw data in Graphpad Prism and Excel file format for all figures are included in S1 Supporting Information.

## Results

### Acute A769662 application suppresses action potential firing in TG neurons

**Fig 1:**
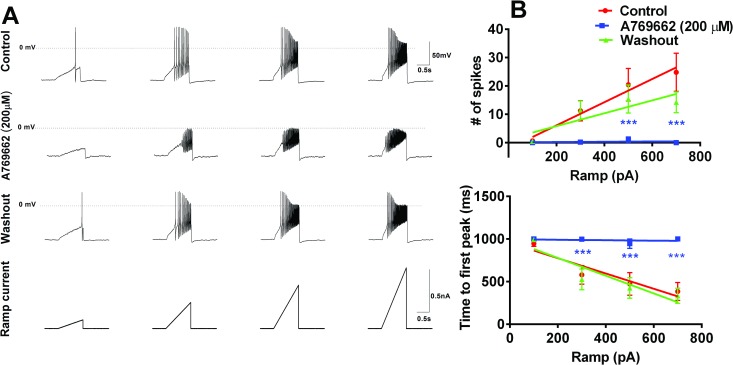
A769662 applied acutely to rat TG neurons blocks AP firing evoked by ramp current stimuli. (A) Representative action potential traces in response to ramp current stimuli before and after acute application of (5 sec) of A769662 (200 μM). Acute application of A769662 (n = 5) onto rat TG neurons significantly reduced numbers of action potentials (B) and reversed time-to-first action potential (AP) peak compared to control (n = 5). This effect was reversible with washout (n = 5). Differences in the mean numbers of action potentials among groups were analyzed by comparing the slopes and intercepts generated from linear regression Comparisons among groups for time to first spike were performed by two-way ANOVA. Colored stars denote significant effects compared to control group. * p < 0.05, ** p < 0.01 and *** p < 0.001.

Nav1.7 is a threshold channel with a slow closed-state inactivation kinetics which make it a primary driver of currents in response to slowly depolarizing ramp stimulus protocols [12,40,41]. Once threshold is reached, other VGSCs, such as Na_v_1.8 may work concurrently with Na_v_1.7 in generating repetitive firing in sensory neurons (Vasylyev and Waxman J Neurophysiol 2012). To evaluate the acute effect of A769662 on action potential (AP) firing in TG neurons, slow ramp currents from 0.1 to 0.7 nA were injected over 1 sec to mimic slow depolarization. Only cells that fired in response to this protocol were evaluated further. Action potentials were defined as spikes that crossed 0 mV and had an amplitude >40 mV. The acute application (< 5 sec) of A769662 (200 μM) onto TG neurons significantly reduced the number of spikes and prolonged the latency to the first action potential spike in response to ramp currents (Fig 1A and 1B). This effect was likely due to a direct block of VGSCs and not to AMPK activation as the effect was rapidly reversed upon washout, an effect inconsistent with altered kinase activity. Based on this finding, we hypothesized that A769662 had a direct effect on VGSCs in addition to its known mechanism of action as a positive allosteric modulator of AMPK.

### The structurally distinct AMPK activator, PT1, suppresses NGF-induced hyperexcitability in TG neurons independently of VGSC blockade

**Fig 2:**
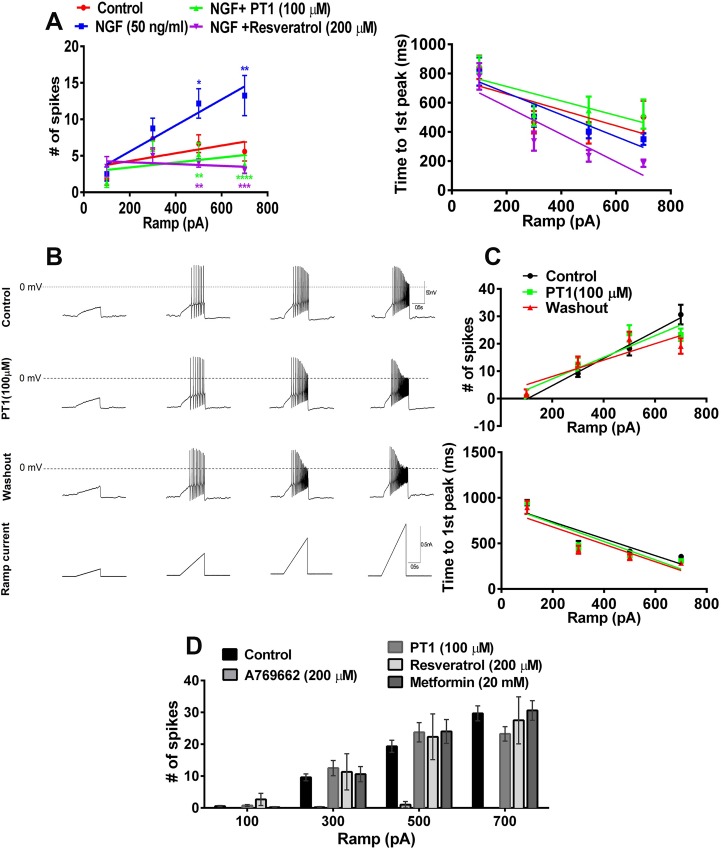
PT1 and resveratrol inhibit NGF-induced hyperexcitability but have no acute effect on action potential firing. (A) Patch clamp analysis of rat TG neurons cultured in the presence of NGF (50 ng/ml, n = 11) show an increase in the number of ramp evoked action potentials compared to control (n = 8) which is reversed by PT1 (100 μM; 1 hr, n = 11) and resveratrol (200 μM, 1 hr, n = 6). Colored stars denote significant effects compared to NGF group. * p < 0.05, ** p < 0.01, *** p < 0.001 and **** p < 0.0001. (B) and (C) Acute application of PT1 (30 μM, n = 10; 100 μM, n = 8) demonstrates no influence on action potential firing compared to control. The number of ramp current evoked action potentials was normalized to control. (D) Acute application of other AMPK activators (resveratrol, 200 μM, n = 6; metformin, 20 mM, n = 6) does not block VGSCs.

PT1 is a small molecule activator of AMPK with a distinct structure and mechanism of action from A769662. It was identified from a screen assessing the activation of a truncated version of the α-subunit of the heterotrimeric AMPK complex composed of the catalytic domain and the autoinhibitory domain (AID; [42]. PT1 is thought to directly disrupt the interaction between the AID and the catalytic domain on the α-subunit to stabilize an active conformation, even in the absence of the regulatory domains [42]. Stimulation of rat TG neurons with depolarizing ramp current injections in the presence or absence of NGF showed that NGF increases TG neuron excitability (Fig 2A). Treatment with PT1 (30 μM, 100 μM) for 1 hr concentration-dependently reduced the number of action potentials fired in response to ramp currents in TG neurons rendered hyperexcitable by NGF exposure (Fig 2A). This finding is consistent with previous results with a diverse set of AMPK activators including A769662 and metformin [33,43]. Another structurally distinct AMPK activator that we have previously shown to be efficacious in preclinical post-surgical pain models is resveratrol [34]. Like PT1, 1-hour treatment with resveratrol also reduced ramp-current evoked spiking in NGF stimulated TG neurons. These findings demonstrate a robust decrease in excitability in TG neurons when exposed to AMPK activators for extended durations of time. An important question is whether the direct VGSC blocking effects of A769662 are unique to A769662 or common to other AMPK activators. In contrast to results obtained with A769662 above, an acute application (<5 sec) of PT1 (100 μM) onto TG neurons without NGF treatment did not alter action potential firing when compared to control (Fig 2B and 2C). These findings suggest that the effects observed with PT1 are not due to a direct channel blocking effect but are likely mediated via a trophic action linked to AMPK activation. Likewise, neither resveratrol (200 μM) nor metformin (20 mM) had an acute effect (< 5 sec application) on ramp current evoked spiking in TG neurons (Fig 2D).

### A769662 rapidly reduces TG neuron Na^+^ current amplitude and lacks use-dependent inhibition

**Fig 3:**
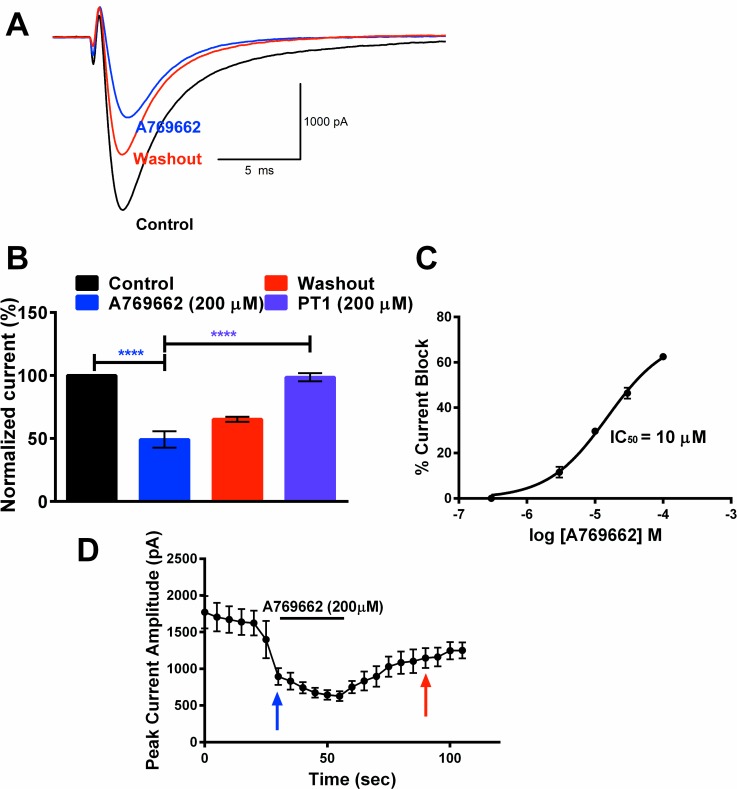
A769662 dose-dependently blocks rat TG VGSC current amplitude. (A) Representative Na^+^ current traces in TG neurons in the presence of A769662. Currents were elicited in TG neurons with a current step protocol initiated from -80 mV to 0 mV for 25 msec from a holding potential of -80 mV. (B) Acute application of A769662 reduced the Na^+^ current amplitude in TG neurons (n = 6, **** p < 0.0001). C) Data generated was fitted to a hill equation to plot the dose response curve for percent current amplitude block by A769662. A769662 inhibited Na^+^ current amplitude with an IC_50_ of 10 μM. D) Peak current vs time plot of the effect of A769662 on Na^+^ current amplitude. Arrows correspond to A769662 (blue) and washout (red) in A) and B).

The current-clamp experiments above suggest a direction action of A769662 on VGSCs. To test this in more detail, small diameter TG neurons (17–20 pF) were voltage clamped in whole-cell configuration and VGSC currents were evoked by a depolarizing test pulse to 0 mV from a holding potential of -80 mV. Currents were evoked by a protocol using 25 ms test pulses delivered every 5 sec (Fig 3A and 3B). Bath application of A769662 (200 μM) rapidly reduced normalized peak sodium currents to 51% ± 6.2 (p < 0.0001). PT1 (200 μM) had no effect on VGSC currents in this experimental paradigm (Fig 3B). The reduction in VGSC currents by A769662 was dose-dependent with an IC_50_ of 10 μM (Fig 3C).

**Fig 4:**
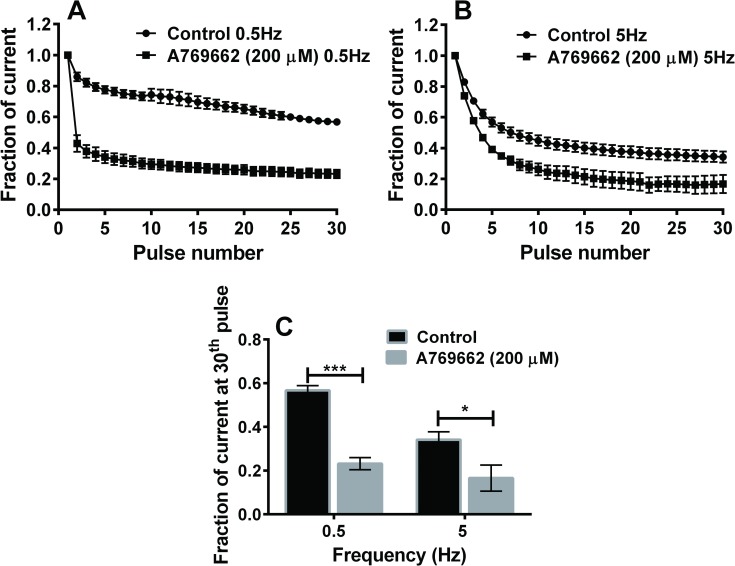
Lack of use-dependent effects of A769662 on rat TG neurons. 30 repetitive 25 ms depolarizing pulses of -10 mV were applied from a holding potential of -80 mV at 0.5Hz (A) and 5Hz (B) in the absence (●) and presence (■) of A769662 (200 μM). Peak current amplitude at each pulse was normalized by the peak current amplitude of the first pulse under each condition and plotted vs the pulse number. (C) Fraction of current at the 30^th^ depolarizing pulse at 0.5 Hz and 5 Hz in the absence and presence of A769662 (n = 5–15, two-way ANOVA, * p < 0.05, *** p <0.001).

Based on the acute block of VGSC currents, we examined whether use-dependent inhibition was present with A769662 to gain further insight into the mechanism of inhibition by A769662. Current amplitudes were measured when elicited by 30 repetitive 25-ms depolarizing pulses to -10 mV from a holding potential of -80 mV at 0.5 Hz and 5 Hz frequencies in the absence and presence of A769662 (Fig 4A and 4B). Under control conditions (without A769662), there was a reduction in the fraction of current at pulse 30 compared to pulse 1 using both frequency protocols (Fig 4C). However, there was a greater reduction at pulse 30 using the 5 Hz protocol when compared to the reduction at pulse 30 using 0.5 Hz, demonstrating frequency-dependent reduction of current in the absence of drug. In the presence of A769662, there was a significant reduction in the amplitude of current at the 30^th^ pulse compared to control using both 0.5 Hz and 5 Hz protocols. However, the fraction of current remaining with A769662 at the 30^th^ pulse at 5 Hz was not significantly different from the fraction remaining at 0.5 Hz (Fig 4C). In the presence of A769662, the higher frequency (5 Hz) failed to evoke a greater reduction in sodium current block when compared to the lower frequency (0.5 Hz) at the 30^th^ pulse. This indicates a lack of use-dependent inhibition of VGSC current by A769662.

### A769662 decreases hNa_v_1.7 currents

**Fig 5:**
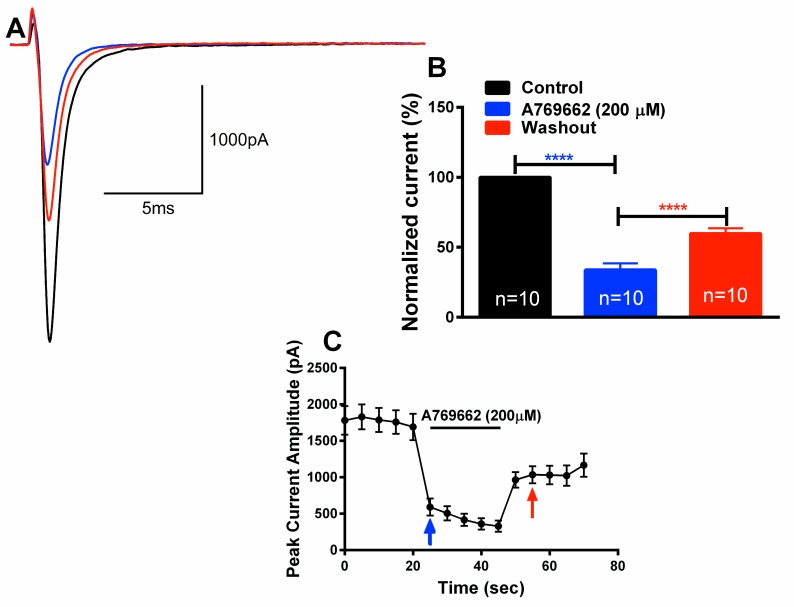
A769662 blocks hNa_v_1.7 currents in HEK cells. (A) A step from -80 mV to 0 mV for 25 ms was used to elicit currents in hNa_v_1.7-transfected HEK cells with or without A769662 application. (B) Acute application of A769662 (200 μM) reduced the Na^+^ current amplitude in hNa_v_1.7-transfected HEK cells (n = 10, one-way ANOVA, **** p < 0.0001 C) Peak current vs time plot of the effect of A769662 on hNa_v_1.7 currents. Arrows correspond to A769662 (blue) and washout (red) in A) and B).

A769662 acutely reduced action potential generation and time to first action potential in TG neurons stimulated with ramp currents. We therefore hypothesized that A769662 might have a direct channel blocking/modulating action on this VGSC subtype. To address this question we used stably transfected Na_v_1.7-HEK cells. Using the same voltage protocol described above, Na_v_1.7 currents were recorded in the absence and presence of A769662 (200 μM). With acute application of A769662, we observed a significant decrease of ~70% in normalized peak Na^+^ current amplitude that washed out over a time course of 40 sec (Fig 5A and 5B).

**Fig 6:**
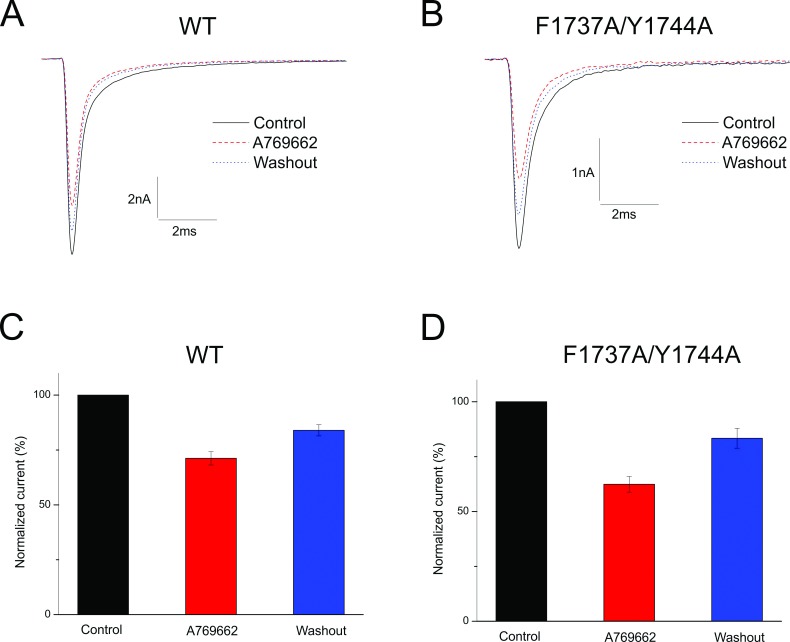
A769662 showed no binding at the local anesthetic binding site. (**A, B**) Nav1.7 current traces recorded under control (solid line), presence of 200μM A769662 (dash line), and washout (dot lines) conditions respectively from a cell expressing WT Nav1.7 channels (**A**) or a cell expressing F1737A/Y1744A mutant channels (**B**); (**C,D**) 200μM A769662 reduced Nav1.7 sodium channel currents from cells expressing WT channels (**C**) and cells expressing F1737A/Y1744A mutant channels (**D**), and washing out could partially reverse the inhibition effects for both WT and F1737A/Y1744A mutant channels.

To elucidate the possible binding site of A769662, we tested the effect of A769662 on the local anesthetic (LA) binding site of sodium channels using a local anesthetic mutant of Na_v_1.7 (F1737A/Y1744A) transiently transfected into HEK293 cells (Fig 6). This double mutant reduces the affinity of Na_v_1.7 to tetracaine >100-fold [44,45]. The block of WT and F1737A/Y1744A channels by A769662 was assayed by measuring the reduction of peak current elicited by depolarizing from - 120 to - 10 mV at a stimulation frequency of 0.2 Hz. With acute application of A769662, Na_v_1.7 sodium currents were reduced by 29% ± 3.0% in WT (n = 10) (Fig 6A and 6C) and 38% ± 3.6% (n = 8, p > 0.05) in F1737A/Y1744A mutant channels (Fig 6B and 6D). Meanwhile the inhibitory effects were partially reversed by washing out for both WT (84 ± 2.5%, n = 10) and F1737A/Y1744A (83 ± 4.6%, n = 8) mutant channels. This finding demonstrates a lack of effect of the double mutant on block of the channel indicating that A769662 is unlikely to bind at the local anesthetic binding site.

### Popliteal fossa administration of A769662 attenuates noxious thermal responses

**Fig 7:**
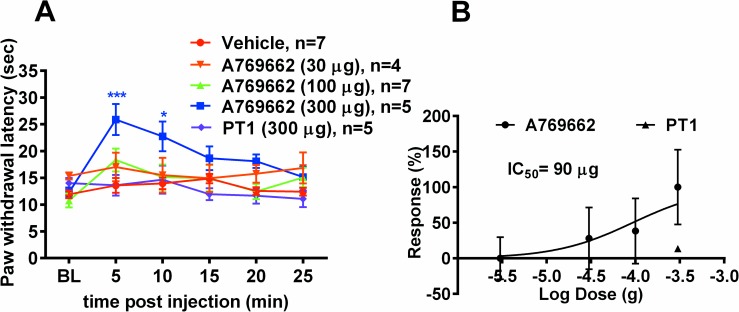
A769662 exhibits local anesthetic effects in rats. (A) Administration of A769662 (30 μg, 100 μg and 300 μg) into the popliteal fossa produces a dose-dependent nerve block by reversing the paw withdrawal latency to noxious thermal heat using the Hargreaves method compared to vehicle treated rats (n = 4–7, regular two-way ANOVA, * p < 0.05 and *** p < 0.001). (B) Paw withdrawal latency plotted as a function of A769662 or PT1 dose. Dose-response curve of A769662 was generated by fitting data to the Hill equation yielding an IC_50_ of 90 μg.

Based on the results presented above, we hypothesized that A769662, but not other AMPK activators, should be capable of eliciting a local anesthetic effect *in vivo* due to its acute VGSC blocking properties. To assess this, we injected A769662 or PT1 into the popliteal fossa and measured noxious thermal hind paw responses using the Hargreaves method. Since popliteal fossa injections were done under light anesthesia, we evaluated the effect of isoflurane on the rotarod to ensure that isoflurane was not having any negative impact on motor performance. Rotarod testing was assessed ~2.5 mins after isoflurane administration and showed no significant difference in latency to fall between naïve rats (no isoflurane, 180s, n = 6) and control rats (with isoflurane induction, 180s, n = 6). There was no standard error in both groups since all the rats in both groups reached cutoff time. Administration of A769662 into the popliteal fossa reduced thermal noxious responses with a rapid onset (peak at 5 min, Fig 7A). This effect was reversible and short lasting. The effect of A769662 was dose-dependent with an IC_50_ of 90 ± 0.212 μg (Fig 7B). Popliteal fossa injection of PT1 did not alter thermal responses at any time point after injection at a dose equal to the maximally effective dose of A769662 (Fig 7A and 7B). Thermal latency was also assessed in the absence and presence of isoflurane induction at the BL, 5,10and 15 mins timepoints. There was no significant difference in thermal latencies between the naïve rats (no isoflurane) and rats with isoflurane induction at any timepoint after anesthesia compared to the naïve condition.(Naïve- BL = 12.24±0.66 sec, 5 min = 11.93±0.90 sec, 10 min = 13.34±0.65 sec,15 min = 13.36±0.91 sec; Control- BL = 13.14±0.23 sec, 5 min = 10.90±0.68 sec, 10 min = 11.44±0.63 sec, 15 min = 12.64±0.78 sec; N = 6 for each group)

## Discussion

We show a novel pharmacological action for the allosteric AMPK activator A769662: inhibition of VGSCs, including human Na_v_1.7. We have investigated a variety of AMPK activating compounds, including other positive allosteric AMPK activators, and this is a unique property of A769662. Because we have previously demonstrated that AMPK activators, including A769662, reduce pathological pain in neuropathic and post-surgical pain models [33,34], these findings create an opportunity for novel dual pharmacology involving activation of AMPK and inhibition of VGSCs by the same molecule.

AMPK has emerged as an important target for neuropathic and post-surgical pain based on the ability of a wide range of AMPK activators to decrease mechanical hypersensitivity and other behavioral measures of pain in these models [33,34,43,46,47,48,49,50,51,52,53,54]. Another widely employed mechanism to produce antinociception in animal models of chronic pain is inhibition of VGSCs that are highly expressed in nociceptors, including Na_v_1.7 and Na_v_1.8 [28,29,55,56]. Na_v_1.7 has risen to a prominent position in the field of analgesic drug development due to genetic findings in patients that show a clear link to this channel in pain regulation in humans [5,8,57,58,59,60]. Our findings show that A769662 inhibits VGSCs, including Na_v_1.7 although it is unlikely that this is the only VGSC inhibited by this compound. Nonetheless, this molecule is a first in class compound that both potently and allosterically activates AMPK and inhibits VGSCs. A769662 is among the most potent AMPK activators described to date, however, its potency at native TG VGSCs is less than that of several specific inhibitors of either Na_v_1.8 or Na_v_1.7 [20,28,29,55]. Hence, this novel pharmacological property of A769662 may be best viewed as an opportunity for further development of this scaffold for more potent modulation of both of these mechanisms of action in a single molecule. Based on the existing evidence for dual mechanism of action, we propose that an optimized compound based on pharmacological properties we have discovered in A769662 would be a powerful modulator of sensory neuron excitability over a long time course.

Relative to other VGSC subtypes, Na_v_1.7 conducts significant current in response to ramp current depolarizations suggesting that Na_v_1.7 may modulate firing thresholds by amplifying generator potentials [12]. Here, we show that A769662 significantly attenuates neuronal firing in response to ramp currents in TG neurons. A769662 produces an acute block of sodium current amplitudes in TG neurons and in stably transfected Na_v_1.7-HEK cells, suggesting VGSC block as a mechanism by which action potential firing is decreased. The lack of use-dependent inhibition and lack of binding at the local anesthetic site by A769662 indicates that A769662 might be acting through a more complex mechanism. Further studies, possibly involving mutations at other sites on the sodium channel are needed to determine the mechanism/location of inhibition employed by A769662. Nevertheless, A769662 was found to have a local anesthetic effect in rats indicating that this compound can reach its site of action on VGSCs *in vivo*. Importantly, a structurally dissimilar AMPK activator did not acutely inhibit VGSCs and had no local anesthetic effect indicating that metabotropic actions of these compounds do not contribute to inhibition of VGSCs or anesthesia over this time course and that the unique effects of A769662 are mediated by VGSC block.

Previous studies demonstrated that a broad variety of AMPK activators decrease neuropathic and post-surgical pain in mice and rat models [33,34,43,48,49,50,51,52,53,54]. The current finding with A769662 could imply that part of this efficacy arises from VGSC blockade. Although we cannot rule out a possible contribution of this mechanism of action, it is very likely that AMPK activation contributes strongly to the behavioral effects of this compound in chronic pain models. The most obvious reason for this is we show here that many other AMPK activators previously demonstrated to profoundly decrease TG neuron excitability have no acute effect on VGSCs. Another piece of evidence is that the acute analgesic effects of A769662 when given into the popliteal fossa were fairly short-lived (on the order of 15 min) indicating that at time points where pain behaviors have previously been measured, any sodium channel block would likely have worn off. In spite of this, our current work suggests that it is possible to develop dual acting compounds that both activate AMPK and inhibit VGSCs. Previous studies have shown long-term effects of systemically administered VGSCs blockers in neuropathic models [61,62,63,64]. Hence, these potential dual acting compounds can be further exploited to extend the duration of action on VGSCs in addition to their AMPK activation activity. Development of compounds with extended durations of action against VGSCs in addition to their AMPK activation activity may have enhanced efficacy over selective AMPK activators alone.

A previous study has suggested that resveratrol blocks VGSCs in native sensory neurons [65]. This finding is in contrast to the data we report here with brief, acute application of resveratrol. Importantly, this previous work applied resveratrol for ~ 10 min before recording VGSC current activity. We have previously shown that resveratrol can activate AMPK on this time scale in TG and DRG neurons from mice [34]. We also show here that prolonged resveratrol application profoundly decreases rat TG neuron excitability, presumably via activation of AMPK since the compound had no effect with acute application. Therefore, a possible explanation underlying these discrepant findings is that AMPK activation was responsible for the altered VGSC currents previously observed with ~ 10 min application of compound. From a mechanistic standpoint, because AMPK activation can modulate MAPK activity in neurons [34] and MAPKs are known to directly phosphorylate VGSCs and increase their excitability [31], this effect on VGSCs seen with prolonged application of resveratrol is likely explained by post-translational modification of VGSCs.

In conclusion, we have discovered a novel dual action of A769662, allosteric AMPK activation and inhibition of VGSCs. Because this dual action covers two important mechanisms of action for the development of analgesic drugs we posit that this finding may serve as a foundation for further development of more potent dually acting compounds. This new class of molecule may have great potential for the treatment of disorders like neuropathic and post-surgical pain where both AMPK and VGSCs have been implicated as key targets [33,34,43,48,49,50,51,52,53,54].

## Supporting Information

S1 Supporting Information Raw data in Graphpad Prism and Excel file format for all figures are included in Supporting Information as SFig1Data–SFig7Data. (ZIP)

## Abbreviations

VGSC: Voltage-gated sodium channel
AP: action potential
AMPK: AMP-activated protein kinase
NGF: nerve growth factor
ERK: extracellular signal-regulated protein kinase
DRG: dorsal root ganglion
TG: trigeminal ganglion
HEK: human embryonic kidney
FBS: fetal bovine serum
LA: local anesthetic
ANOVA: analysis of variance
